# EEG oscillatory changes following rehabilitative tactile and thermal stimulation in disorders of consciousness: a longitudinal and pilot study

**DOI:** 10.3389/fnins.2026.1855580

**Published:** 2026-07-28

**Authors:** Caterina Formica, Simona De Salvo, Elvira Gjonaj, Serena Dattola, Angelo Quartarone, Antonella Alagna, Jolanda De Caro, Anna Lisa Logiudice, Carmela Rifici, Silvia Marino

**Affiliations:** IRCCS Centro Neurolesi Bonino Pulejo, Messina, Italy

**Keywords:** disorder of consciousness, electroencephalogram, frequency oscillation, rehabilitation, sensory stimulation

## Abstract

**Introduction:**

Recent advances in the understanding of sensory processing in disorders of consciousness (DoC) have highlighted the potential of exteroceptive stimuli, particularly tactile stimulation. Unlike auditory or visual inputs, tactile stimuli engage primary somatosensory pathways that may be preserved even in severely brain injured patients. The aim of our study is to identify cortical neurophysiological markers for diagnosis, prognosis, and structured rehabilitative planning.

**Methods:**

Seven patients were included. EEG was recorded at baseline (T0) and after 1 month of repeated tactile and thermal stimulation administered in a randomized manner to both hands (T1). Spectral power changes were computed by contrasting stimulation-related activity with resting state and analyzed across frequency bands and cortical regions.

**Results:**

At T0, sensory stimulation elicited weak and spatially limited EEG modulation, predominantly involving slow-frequency activity, consistent with reduced cortical responsiveness. Following the stimulation period (T1), EEG responses showed increased amplitude and spatial extent, characterized by a relative suppression of slow oscillations and enhanced modulation of higher-frequency activity over fronto-central and parietal regions.

**Conclusion:**

Repeated tactile and thermal stimulation were associated with longitudinal changes in EEG oscillatory patterns, reflecting possible modulation of cortical responsiveness associated with sensory stimulation. The assessment of stimulation-induced EEG oscillatory changes may represent exploratory neurophysiological indicators of residual cortical responsiveness that could support future studies on sensory processing in DoC patients.

## Introduction

Disorders of consciousness (DoC), including the vegetative state/unresponsive wakefulness syndrome (VS/UWS) and the minimally conscious state (MCS), represent some of the most challenging conditions in clinical neuroscience. Accurate diagnosis and prognosis in DoC are critical due to the complex interaction between preserved neural networks and impaired behavioral responsiveness (Porcaro et al., 2022). In recent years, neuroimaging studies on DoC provided different assumptions: it was found that they could maintain varying cognitive reserves that are not assumed through behavioral patterns (Berlingeri et al., 2019). Marino et al. (2017) demonstrated that functional connectivity is significantly disrupted the default mode network (DMN). In patients with VS and MCS, the integrity of the DMN correlates with the level of consciousness also to provide a promising result for diagnosis and prognosis of these patients (Marino et al., 2017). These findings have significant clinical implications especially in the early stage in which the alterations of consciousness are variable; highlighting the importance of differential diagnosis between a VS/UWS and a MCS for accurate prognostic information and structured specific therapeutic approaches (Le Cause et al., 2024). In recent years, several studies have examined the residual sensory processing in DoC patients by using neuroimaging and neurophysiological methods, as well as the Event-Related Potentials (ERPs) measurement (De Salvo et al., 2015a,b) including assessment of the nociceptive system through painful laser stimulation. Laser evoked potentials (LEPs) could be considered a valid and feasible method to evaluate the nociceptive and somatosensory pathway, showing a distributed cortical network activation including the thalamus, insula, anterior cingulate cortex, and fronto-parietal associative regions (Bonin et al., 2023), even in patients with severely impaired behavioral responsiveness. The preservation of these cortical responses has been interpreted as a marker of residual thalamo-cortical connectivity and covert sensory processing, suggesting that cortical responsiveness to external stimulation may persist despite limited overt behavioral interaction (Boly et al., 2008; De Salvo et al., 2015b). These findings highlighted the growing importance of integrating neuroimaging and neurophysiological assessments into clinical practice to improve diagnostic accuracy, reduce the risk of misdiagnosis, and more recently effective rehabilitative treatment approaches for DoC patients. Recent advances in the understanding of sensory processing in DoC have highlighted the potential of exteroceptive stimuli, particularly tactile stimulation. Unlike auditory or visual inputs, tactile stimuli engage primary somatosensory pathways that may be preserved even in severely brain injured patients. First, a tactile stimulus indicates a direct possible danger; second, it is important for social human interaction. Immediate responses to affective touch and gentle stroking are mediated by specialized low-threshold mechanoreceptors linked to unmyelinated C-tactile (CT) fibers. These receptors are associated with primary and secondary somatosensory cortices which are also involved in emotional arousal (McGlone et al., 2014). Previous studies showed that DoC patients could respond to tactile stimulation and changes were observed in EEG patterns (Laureys, 2005; Portnova et al., 2020). Moreover, thermal stimulation has also been associated with dynamic modulation of EEG signals, supporting the sensitivity of oscillatory activity to somatosensory and nociceptive processing (Mulders et al., 2020). Similar modulatory effects have also been reported for tactile stimulation in patients with disorders of consciousness. It was observed that theta rhythm power decreased during tactile stimulation in DoC patients compared to the control group; these findings correlated positively with coma recovery outcome and gray matter volume in the right putamen and insula, while an improvement in beta band power correlated with the thickness of somatosensory cortices (Portnova et al., 2020). Emerging evidence suggested that tactile paradigms can elicit measurable EEG responses, such as ERPs, which may reflect preserved thalamo-cortical connectivity and higher-order cortical processing (Hoefer et al., 2016; Pauzin and Krieger, 2018). Neuroimaging studies highlighted that CT fiber stimulation activates posterior insula; therefore, perception of tactile stimuli includes the involvement of prefrontal areas, cingulate cortex and posterior superior temporal sulcus (Chen et al., 2025; Rolls et al., 2003). In summary, the stimulation of CT fibers engages neural networks implicated in emotional and cognitive processing. The activation of these areas may provide novel insights into the functioning of patients in a DoC state, through a form of stimulation that is both non-invasive and easy-to-use (Bonanno et al., 2025). However, lack of standardized protocols of sensory stimulations and neurophysiological markers limited their use in clinical and rehabilitative program. This study aims to identify EEG correlates of cortical responsiveness associated with tactile and thermal stimulation in DoC patients.

## Materials and methods

### Participants

A total of 7 right-handed patients (5 males, 2 females; mean age: 50.3 ± 8.2 years) were included in the study. All individuals had transitioned from a VS to a MCS and remained in MCS at the time of assessment. The average duration of the coma phase was 1.6 ± 0.7 months, followed by a vegetative state lasting on average 2.5 ± 1.2 months prior to the emergence of MCS. The etiology of stroke was hemorrhagic (4 patients) and ischemic (3 patients). At the time of inclusion, all patients demonstrated a Coma Recovery Scale-Revised (CRS-R) (Giacino et al., 2004) total score of 8, with no significant variation across the sample (Table 1), no other neurological or mental illnesses, as well as signs of epileptic brain activity. The detailed quantitative lesion mapping was not systematically available for all participants.

**TABLE 1:** Demographic and descriptive variables of participants.

Variable	Mean ± SD
Etiology	Hemorrhagic stroke = 4, ischemic stroke = 3
Age (years)	40.3 ± 3.2
Coma phase (months)	1.6 ± 0.7
Time in vegetative state (months)	2.5 ± 1.2
CRS-R score (T0)	8.0 ± 3.23
CRS-R score (T1)	10.28 ± 3.14

SD, standard deviation; CRS-R, Coma Recovery Scale-Revised; T0, baseline assessment; T1, assessment after treatment.

The work was carried out in accordance with The Code of Ethics of the World Medical Association (Declaration of Helsinki) for experiments involving humans. An informed consent was obtained from the legal representatives to participate to the study. The research was approved by the Local Ethic Committee I (code protocol: 09/2018).

### Procedures

**FIGURE 1:**
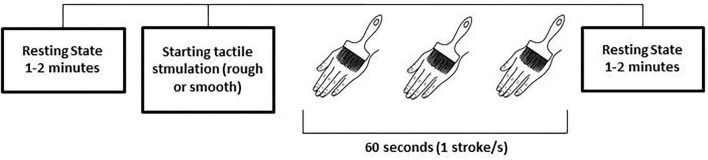
Experimental paradigm for tactile stimulation–slow stroking with brush with velocity 2 cm/s, medium pressure and duration of 60 s. The same paradigm was used for rough and smooth tactile stimulation.

**FIGURE 2:**
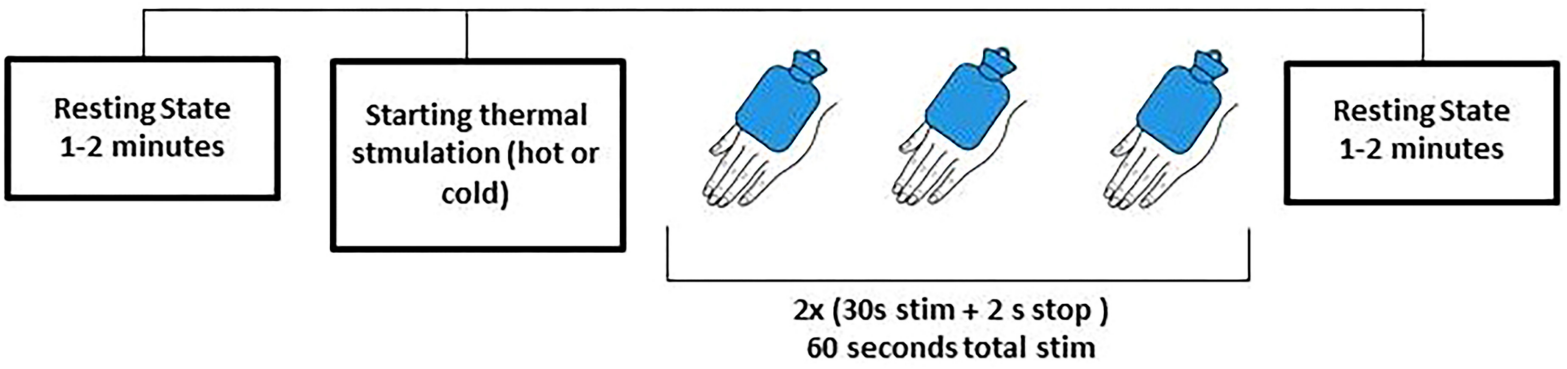
Experimental paradigm for thermal stimulation–placement of cold/hot bag for 30 s, stop of 2 s and repeat for other 30 s, no pressure for a total of 60 s stimulation. The same paradigm was used for hot and cold thermal stimulation.

The study followed a longitudinal within-subject design with two assessment time points. At baseline (T0) the protocol included EEG acquisition in resting state EEG for 5 min and during tactile and thermal stimulation sequences. Each tactile stimulation was performed with a constant velocity that was provided by a visual metronome set at 1 s per stimulus for a total of 60 s equivalent to 60 stimuli. The tactile stimulation was performed with a brush to create a rough sensation, one for smooth sensation and another one to create a soft sensation on the dorsal surface of the hand with a constant velocity of 2–4 cm/s (Figure 1). Thermal stimuli were administered using ice and hot water (30 °C) to induce cold and warm sensory perceptions. The stimuli were applied for 15 consecutive seconds alternating with 2 s of stop for a total of 60 s for each thermal stimulus (Figure 2). After that, for 1 month patients received a sensory stimulation protocol, in which they were stimulated with daily sessions of passive sensory stimulation, including tactile and thermal stimuli. Stimuli were administered in a randomized order and applied bilaterally on both hands to avoid lateralization bias and habituation effects. The protocol was submitted for 5 days a week for 40 min per session. T1 was recorded after 1 month of daily sensory stimulation with the same modality of baseline recording.

### EEG data analysis

Each patient underwent a 5-min resting-state EEG recording, followed by EEG acquisition during a stimulation protocol including tactile (smooth, soft, and rough) and thermal (cold and hot) stimuli, performed both before (T0) and after (T1) stimulation protocol. EEG signals were recorded using a Brain-Quick System (Micromed) with 19 scalp electrodes positioned according to the international 10–20 system (Fp1, Fp2, F7, F8, F3, F4, Fz, T3, T4, C3, C4, Cz, P3, P4, Pz, T5, T6, O1, O2). Recordings were acquired in a differential montage referenced to linked earlobes. Electrode impedances were maintained below 5 kΩ. Data were sampled at 256 Hz. EEG preprocessing was performed offline using EEGLAB (v2025.1.0) and custom MATLAB (R2023b) scripts. A 50-Hz notch filter was applied to reduce line noise, and data were band-pass filtered between 1 and 40 Hz. Bad channels and artifactual segments were automatically identified and removed before applying independent component analysis (ICA), which was performed on average-referenced data. ICA components classified as ocular or muscular artifacts with a probability greater than 0.9 were automatically rejected. Removed channels were subsequently interpolated, and the final EEG signals were visually inspected to ensure adequate data quality before further analysis. Power spectral density (PSD) was estimated over 1-min EEG segments using Welch’s method with 2-s Hamming windows and 50% overlap, providing a stable estimate of the spectral content for each electrode. EEG band power was then calculated by integrating the PSD over the following frequency ranges: delta (1–4 Hz), theta (4–8 Hz), alpha (8–13 Hz), beta (13–30 Hz), and gamma (30–40 Hz).

## Results

To investigate longitudinal changes in cortical responsiveness to sensory stimulation, EEG spectral power during each stimulation condition was compared with the corresponding resting baseline for each subject. Specifically, for each electrode, frequency band, stimulation side, and sensory condition, the difference between spectral power during stimulation and baseline was calculated. This difference reflects the extent to which sensory stimulation modulated EEG activity relative to resting conditions. These stimulation-related power changes were then compared between T0 and T1 using two-tailed Wilcoxon signed-rank tests (*p* < 0.05). Given the large number of statistical comparisons performed across electrodes, frequency bands, stimulation sides, and sensory conditions, false discovery rate (FDR) correction was applied to control for multiple comparisons. No results survived FDR correction; therefore, the uncorrected findings reported below should be interpreted as exploratory patterns rather than statistically confirmed effects. Overall, greater stimulus-related EEG power at T1 compared with T0 was most consistently observed during right-sided stimulation in the beta and gamma frequency bands. During both thermal stimulations, these increases were mainly observed over frontal and occipital regions, with additional temporal involvement for the hot condition. Similar patterns were observed for tactile stimulation: the smooth condition showed beta-band increases over frontal and temporal electrodes and gamma-band increases over frontal and occipital areas, while the soft condition exhibited overlapping beta- and gamma-band increases mainly across frontal and occipital electrodes. For the rough condition, increases were primarily localized over occipital regions in both beta and gamma bands, with additional frontal involvement in the gamma band.

**FIGURE 3:**
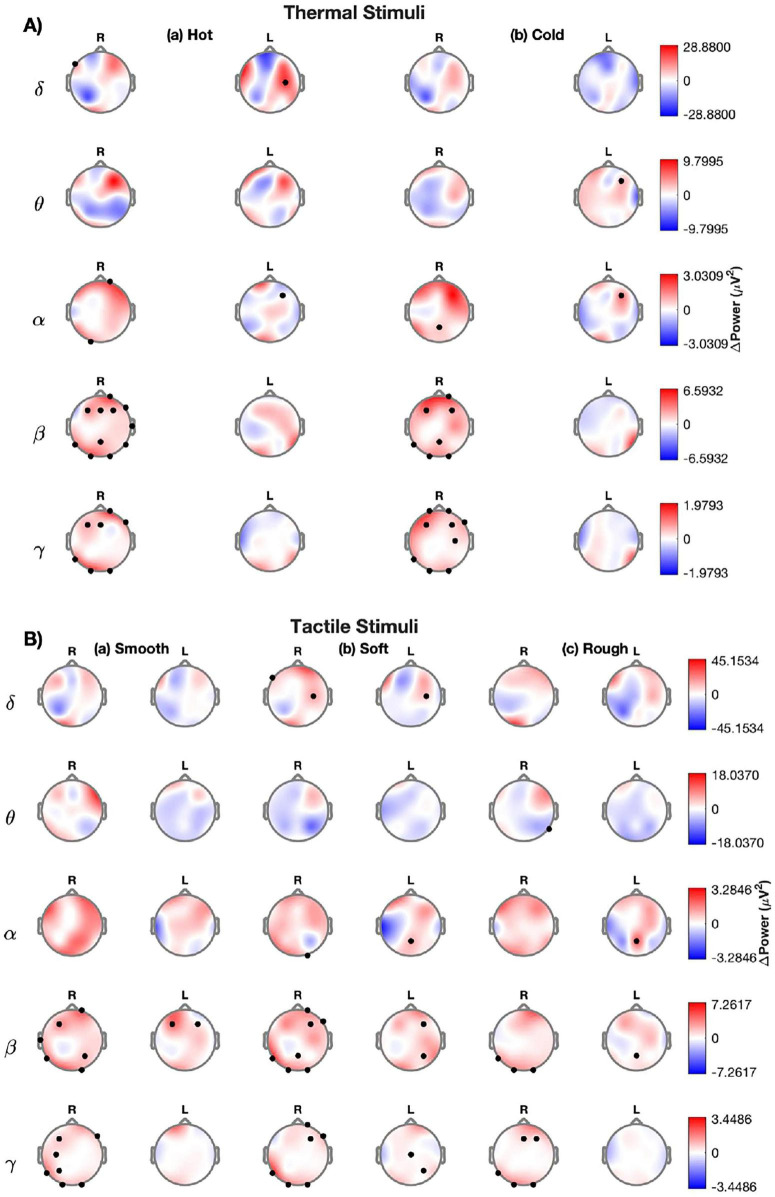
Topographical maps of the difference between median stimulation-related spectral power at post-treatment (T1) and pre-treatment (T0) conditions (ΔPower = median T1 – median T0). Stimulation-related power was obtained for each subject by subtracting resting-state baseline power from power recorded during sensory stimulation. **(A)** Shows thermal stimulation [(a) hot, and (b) cold], **(B)** reports tactile stimulation [(a) smooth, (b) soft, and (c) rough]. Rows correspond to frequency bands (δ, θ, α, β, γ), and columns represent stimulation side (R = right, L = left). Color scales indicate power differences in μV^2^, with red denoting greater stimulation-related power at T1 compared with T0 and blue indicating greater power at T0. Black dots mark electrodes showing statistical significance (*p* < 0.05).

For left-sided stimulation, fewer consistent effects were observed. Specifically, the smooth condition showed beta-band increases at T1 compared with T0 over two frontal electrodes. Additional isolated electrode-level effects were observed but were not spatially consistent. Topographical maps for each condition are presented in Figure 3, while the corresponding bar plots quantifying the differences between T0 and T1 are provided in the Supplementary Figures 1, 2.

## Discussion

The present longitudinal study investigated the EEG oscillatory changes before (T0) and after (T1) 1 month of repeated tactile and thermal stimulations, evaluating neurophysiological cortical markers. The results showed an improvement in EEG responsiveness to the sensory stimulation at T1 across some EEG frequency bands, suggesting an association between repeated sensory stimulation and changes in EEG oscillatory activity. The main finding was an improvement in stimulus-related EEG reactivity at T1 compared with T0, predominantly expressed as enhanced modulation in faster oscillatory activity, particularly within the beta and gamma ranges. The literature has associated spectral EEG patterns in DoC with a reduction in alpha and beta power, together with increased delta activity, as a common feature of severe unresponsiveness and large-scale network breakdown (Lehembre et al., 2012; Sitt et al., 2014). Higher alpha power, reactivity, and complexity metrics correlate with improved outcomes and consciousness levels in minimally conscious and recovering patients, as highlighted in systematic reviews on prognostic EEG biomarkers in DoC rehabilitative settings (Ballanti et al., 2022). Other studies on spectral dynamics highlighted that transitions from VS to MCS involved significant changes in EEG features, including reductions in slow-wave activity and increases in higher frequency band activity linked to richer network interactions (Lei et al., 2022). At T1, we observed a predominance of beta and gamma band modulation, suggesting increased cortical processing complexity. These effects were more consistently observed during right-sided stimulation, involving frontal, temporal, and occipital regions, whereas left-sided stimulation elicited fewer and less spatially consistent responses (Figure 3). Overall, these findings suggested that repeated sensory stimulation may suggest exploratory changes in cortical responsiveness associated with repeated sensory stimulation, potentially reflecting increased engagement of sensory-related neural networks over time. Interpretation of gamma-band findings should be approached cautiously. The low-density EEG montage, the sampling rate, the possible contribution of residual muscular artifacts in the 30–40 Hz range, and the overlap between the analyzed gamma band and the low-pass filter cutoff may have influenced high-frequency activity estimates. Faster oscillatory activity has been associated with sensory-motor integration stimuli and cortico-cortical communication, possibly reflecting modulation of residual cortical responsiveness that may not be detected through behavioral assessment only (Wang et al., 2024). However, the present findings do not allow direct inferences regarding functional recovery or neuroplasticity. Furthermore, previous studies reported that chronic stroke patients exhibit elevated delta/theta and depressed beta activity compared to healthy controls, and that rehabilitation-associated improvements correlate with partial restoration of beta band power and connectivity, reflecting sensorimotor and cognitive reintegration (Zhang et al., 2024). The effects were observed over frontal and parietal–occipital regions, supporting the involvement of associative and higher-order sensory areas; the study revealed the importance of a fronto-parietal network, characterized by a high prevalence of long-range connections (Sikkens et al., 2019). The lateralization of effects toward right-sided stimulation represents an additional finding of potential clinical relevance. Asymmetric EEG responsiveness has been previously reported in patients with severe brain injury and in unresponsive conditions and has been interpreted as reflecting unequal preservation of thalamo-cortical and cortico-cortical dynamics, as well as hemispheric differences in lesion distribution and network integrity (Zheng et al., 2026). In this context, the predominance of stimulus-related oscillatory modulation over the right hemisphere was observed, indicating a greater residual skill of sensory-driven cortical engagement within specific functional networks (Struber et al., 2021). These findings suggested that lateralized EEG responses to sensory stimulation may represent a potential marker of residual cortical reactivity and may contribute to a better characterization of EEG responsiveness patterns during sensory stimulation in DoC patients due to oscillatory remodulation provided by tactile stimulation. However, several limitations should be acknowledged. First, the sample size was relatively small, limiting statistical power, generalizability, and the strength of inferences regarding neuroplasticity, prognosis, or cortical reorganization. Moreover, none of the observed effects survived FDR correction for multiple comparisons; therefore, the findings should be considered exploratory and interpreted with caution. Second, a limitation is the absence of a control condition. However, in patients with DoC, spontaneous neurophysiological changes over such a limited period are generally expected to be minimal in the absence of structured rehabilitative interventions. Therefore, while spontaneous fluctuations cannot be fully excluded, they are not fully account for the observed EEG modifications. Third, detailed information regarding lesion lateralization and quantitative neuroanatomical mapping was not systematically available for all participants. In addition, the analysis was conducted at the level of individual electrodes and spectral power, without assessing functional connectivity or cross-frequency interactions, which could provide further insight into network-level mechanisms. Despite these limitations, some strengths should be considered, such as the longitudinal within-subject design, the multimodal sensory stimulation paradigm, and the comprehensive spectral analysis across frequency bands relevant to consciousness and network integrity.

## Conclusion

In conclusion, the present findings indicate that repeated tactile and thermal stimulation is associated with longitudinal modulation of EEG oscillatory activity in patients with DoC, characterized by increased stimulus-related reactivity and a shift toward faster cortical dynamics. These EEG changes may reflect modulation of cortical responsiveness associated with repeated sensory stimulation, suggesting the potential value of stimulation-induced EEG measures as exploratory tools for monitoring cortical function and potentially supporting diagnostic evaluation and individualized rehabilitation planning in this clinically challenging population.

## Data Availability

The raw data supporting the conclusions of this article will be made available by the authors, without undue reservation.

## Supplementary material

Supplementary Figure 1 Bar plots of thermal stimulation-related EEG spectral power differences between post-treatment (T1) and pre-treatment (T0) conditions (ΔPower = T1 − T0) for each electrode. Values represent the difference between the median stimulation-related power at T1 and T0, where stimulation-related power was obtained by subtracting resting-state baseline power from stimulation power for each subject. **(A)** Shows right-sided stimulation, while **(B)** shows left-sided stimulation. Columns correspond to sensory conditions [(a) hot, and (b) cold], and rows to frequency bands (δ, θ, α, β, γ). Positive values indicate greater stimulation-related power at T1 relative to T0. Asterisks denote electrodes showing statistically significant differences between sessions (*p* < 0.05).

Supplementary Figure 2 Bar plots of tactile stimulation-related EEG spectral power differences between post-treatment (T1) and pre-treatment (T0) conditions (ΔPower = T1 − T0) for each electrode. Values represent the difference between the median stimulation-related power at T1 and T0, where stimulation-related power was obtained by subtracting resting-state baseline power from stimulation power for each subject. **(A)** Shows right-sided stimulation, while **(B)** shows left-sided stimulation. Columns correspond to sensory conditions [(a) smooth, (b) soft, and (c) rough], and rows to frequency bands (δ, θ, α, β, γ). Positive values indicate greater stimulation-related power at T1 relative to T0. Asterisks denote electrodes showing statistically significant differences between sessions (*p* < 0.05).
